# Diagnosis of Overtraining Syndrome: Results of the Endocrine and Metabolic Responses on Overtraining Syndrome Study: EROS-DIAGNOSIS

**DOI:** 10.1155/2020/3937819

**Published:** 2020-04-22

**Authors:** Flavio Adsuara Cadegiani, Pedro Henrique Luiz da Silva, Tatiana Camargo Pereira Abrao, Claudio Elias Kater

**Affiliations:** Adrenal and Hypertension Unit, Division of Endocrinology and Metabolism, Department of Medicine, Escola Paulista de Medicina, Universidade Federal de São Paulo (EPM/UNIFESP), São Paulo, SP, Brazil

## Abstract

**Objectives:**

Overtraining syndrome (OTS), a common dysfunction among elite athletes, causes decreased performance and fatigue and has no standardized diagnostic criteria. The Endocrine and Metabolic Responses on Overtraining Syndrome (EROS) study identified more than 45 potential biomarkers of OTS. In the present study, we hypothesized that combinations of these biomarkers could be an accurate diagnostic tool for OTS.

**Methods:**

We selected parameters with largest difference and fewest overlapping results compared to healthy athletes and highest feasibility and reproducibility. Among the multiple combinations attempted, we chose those that did not show overlapping results, according to the objective.

**Results:**

We included 11 clinical parameters, 4 basal hormones, and 5 hormonal responses in Insulin Tolerance Test (ITT). The three selected diagnostic tools were the (i) EROS-CLINICAL, with only clinical parameters, which was suitable as an initial assessment for athletes suspected of OTS; (ii) EROS-SIMPLIFIED, with clinical parameters and basal hormones, when the EROS-CLINICAL was inconclusive; and (iii) EROS-COMPLETE, with basal and hormonal responses to stimulation tests, which was valuable for population-based screening, research purposes, and unusual presentations of OTS.

**Conclusion:**

We identified innovative tools with 100% accuracy for the diagnosis of OTS, without the need to exclude confounding disorders.

## 1. Introduction

A persistent imbalance between training and recovery among athletes, which may lead to decreased performance and fatigue, has been termed overtraining syndrome (OTS). Chronic insufficient energy availability and the depletion of repair mechanisms lead to multiple dysfunctional adaptations in hormonal, immune, inflammatory, and metabolic pathways and ultimately to the clinical manifestations of OTS [1–5]. Indeed, despite the periodization of exercises as a tool to prevent OTS, its incidence is likely increasing and expanding to nonelite athletes and may be solely caused by non-exercise-related triggers, including chronic low energy intake and availability, particularly among young female athletes [1–5].

OTS substates include functional overreaching (FOR), when performance decreases for a few days, with further improvement after recovery [1]; nonfunctional overreaching (NFOR), when decreased performance lasts slightly longer than FOR (up to two-three weeks), but ends with a full recovery [1]; and overtraining syndrome (OTS), when decreased performance lasts longer (several months to years) and is accompanied by a severe mood disorder [1]. However, the limits of FOR, NFOR, and OTS are not precise, and the diagnosis of OTS is possible only when athletes do not fully recover after a certain period of rest, for which the period of time required for the diagnosis of OTS varies between three weeks and two months, although a specific point is still disputable [1–6]. Indeed, overreaching and overtraining are considered different states along the same continuum [1, 4, 5]. Although the prevalence of OTS has not been accurately estimated, the intense monitoring tools and the learning of the periodization for the prevention of excessive training may not have been enough to reduce its occurrence, as other triggers including insufficient caloric intake, excessive concurrent cognitive activity and biopsychosocial issues, and bad sleep quality and hygiene were not as surveilled as training intensity, volume, and programming. Indeed, without the control of all these triggers, the prevalence of OTS may increase, owing to the growing numbers of professional and nonprofessional athletes, in addition to the fact that periodization of training sessions is still not unanimous and not always strictly followed by athletes.

The development of accurate definitions and diagnostic criteria for OTS are challenging, due to its complex pathophysiology and individual combinations of dysfunctions found in each OTS-affected athlete [1, 4]. Multiple attempts to identify biomarkers and underlying mechanisms of OTS have been inconclusive [1–4], as reported in a recent systematic review^21^. Therefore, the diagnostic assessment of OTS remains disputable, based on a flowchart with clinical and biochemical exclusion criteria [1–3], as presented in the latest guidelines on OTS [1].

The Endocrine and Metabolic Responses on Overtraining (EROS) study, which was designed to address the aforementioned questions, concurrently compared 117 parameters, including multiple basal and stimulated hormones; inflammatory, muscular, and immune markers; eating, social, and sleeping patterns; psychologic characteristics; and body metabolism and composition, between three study groups in a sample of 51 participants. The three groups consisted of athletes affected by OTS (OTS group), healthy athletes (ATL group), and individuals who served as not physically active controls (NPAC group) [4–7]. Two control groups were used to determine which parameters were overtly affected (altered when compared to both the ATL and NPAC groups), which were relatively affected (altered when compared to the ATL group, but similar to NPAC group), and which were unchanged in OTS (similar to both the ATL and NPAC groups, or similar to the ATL group).

Although more than 40 novel markers were identified [8–11] of OTS, none of them accurately distinguished between OTS-affected athletes and healthy athletes when analyzed individually. Furthermore, choosing the most appropriate markers could cause confusion among practitioners, and the task could be time-consuming and expensive, requiring the knowledge of a specialist healthcare provider.

Conversely, given the characteristics of OTS, we hypothesized that different combinations of OTS biomarkers would yield accurate results for a diagnosis of OTS. Indeed, when certain combinations of parameters were tested, OTS could be accurately identified in all affected athletes while excluding OTS in all healthy athletes, without the need to exclude confounding disorders. The use of combinations of different risk factors facilitated our identification of athletes at risk for OTS, thereby preventing its occurrence. Hence, the objective of the present study was to suggest tools for the early diagnosis and prevention of OTS, using combinations of easy, inexpensive, and highly accurate parameters identified in the EROS study [8–11], for use by a wide range of sports professionals.

## 2. Materials and Methods

The selection process, baseline characteristics, and results of the arms of the EROS study are presented in previously published papers [5–8], and the raw data are available at a repository (https://osf.io/bhpq9/).

We recruited male participants through sports coaches, social media, and group messages (WhatsApp and Telegram). Each candidate provided information regarding age, sex, weight, height, and the group they intended to participate in—if suspected for overtraining syndrome: OTS; if healthy athlete: ATL; and if nonphysically active: NPAC.

Criteria for exclusion were (1) extremes of age (<18 y/o and >50 y/o), (2) false athletes (training <300 minutes/week, < moderate-to-intense intensity, and <6 months consecutively), (3) misdiagnosis of OTS (lack of unexplained decreased performance and presence of confounding clinical, biochemical, or psychiatric dysfunctions that could lead to decreased performance), (4) use of confounding drugs or hormones, and (5) presence of confounding diseases (and altered biochemical or hormonal levels, that may also justify the reduced performance).

A total of 51 participants were selected (OTS = 14; ATL = 25; and NPAC = 12). From these three groups, the two groups of athletes—OTS-affected athletes (OTS) and healthy athletes (ATL group)—were included in the present study, in a total of 39 participants. The control group of nonactive participants was not included because the aim was to distinguish healthy athletes from those at risk for OTS, for which nonactive controls were unnecessary.

In the EROS study, 117 markers in the EROS study were evaluated, including basal and accumulated hormonal levels, hormonal responses to stimulation tests, body metabolism and composition, social and psychological aspects, and specific eating patterns. All the selected biochemical data were determined using standardized commercially available assay kits [8–11] (https://osf.io/bhpq9), with inter- and intra-assay coefficients of variability below 3.5% and 3%, respectively.

Of these parameters, 44 were excluded, including those that were intrinsically linked to other parameters, that by themselves did not determine diagnoses or status, that were invalid and/or unsubstantiated, that did not provide additional independent data, that were qualitative indices, and those that were missing in more than 5% of the participants.

**Figure 1:**
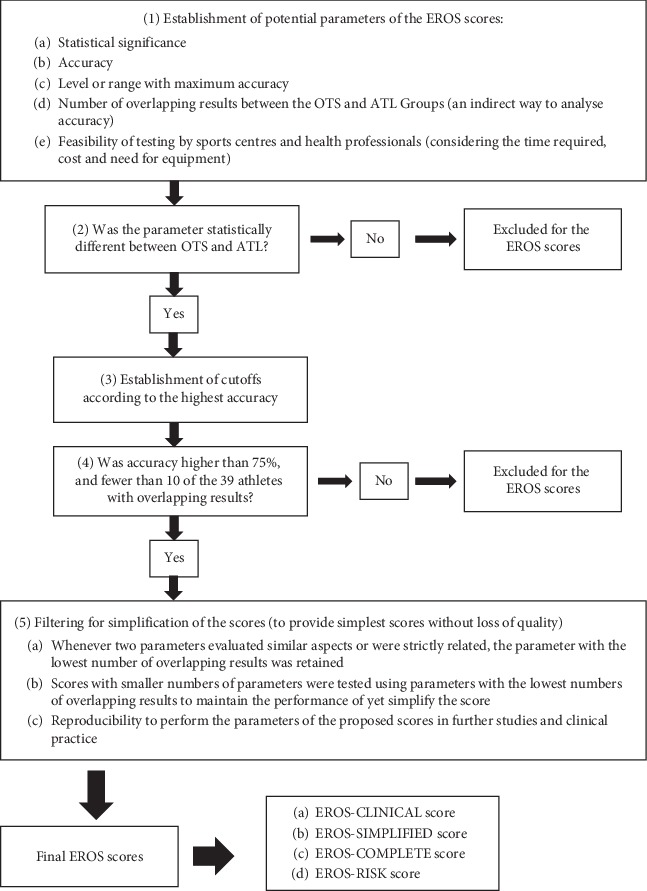
Sequence of steps for the identification of the most appropriate markers for the EROS tools.

At the second step, of the 73 previously selected parameters (listed in Table 1), although none fully distinguished OTS-affected from healthy athletes, those that better identified OTS-affected were selected. The sequence of steps used to identify the most appropriate markers is shown in Figure 1. The following criteria for eligibility were used: (1) quantitative, not qualitative data; (2) variables not inexorably linked to another variable, i.e., interdependent variables (e.g., % of cortisol increase and delta cortisol are inexorably linked variables; therefore, one of them was excluded); (3) validated and standardized tests and parameters; (4) data showing statistically significant differences between OTS and ATL groups (*p* < 0.05); (5) high accuracy (>75%); and (6) few overlapping results between OTS and ATL groups (<10, among 39 athletes). The cutoff for each parameter was based on the highest level of accuracy needed to distinguish the healthy from OTS-affected athletes. Among the parameters that fulfilled the criteria, those that were the easiest to test, the least time-consuming to test, and had the lowest cost were selected, as they increased the likelihood of reproducibility in further studies and clinical practice. The individual examination of each clinical and biochemical parameter for the selection process is detailed in Tables 2 and 3, respectively.

We tested all possible combinations of two or more parameters on two groups of participants (OTS-affected athletes and healthy athletes). We assigned scores to participants for each combination attempt and compared the two groups' results.

First, we selected combinations that did not yield overlapping results between OTS-affected and healthy athletes (i.e., combinations that distinguished each group). Among the selected combinations, we chose tests that were easiest to perform and tests that detected the largest differences between the two groups. These combinations included one with that consisted exclusively of clinical markers, one that combined clinical and basal biochemical parameters, and one that included clinical and basal biochemical markers and stimulated hormones. We referred to these combinations as EROS-CLINICAL, EROS-SIMPLIFIED, and EROS-COMPLETE, respectively. The three combinations were then proposed as diagnostic tools for OTS without the need to exclude confounding disorders.

For the proposed diagnostic tools, we added two hallmark characteristics of OTS that are *sine-qua-non* criteria for its diagnosis: unexplained prolonged decrements in performance and a subjective sense of increased effort during training to achieve the same volume and intensity and unresponsiveness to rest, which was necessary to exclude the diagnosis of overreaching.

Finally, we proposed a fourth combination of parameters, termed EROS-RISK, which included the major risk factors for OTS identified in the EROS study, for the identification of athletes at imminent risk of OTS.

The characteristics of the proposed diagnostic tools are summarized in Table 4.

### 2.1. Statistical Analysis

The analyses of the statistical significance of the parameters evaluated in the EROS study are described in the arms of the EROS study [5–8]. Evaluations of the accuracy, PPV, and NPV were performed to select the most suitable markers using Excel® for Mac (Microsoft Corp., Redmond, WA, USA). The tests of all combinations of parameters, results, and the selection of diagnostic tools were performed using SPSS 24.0 (IBM, USA).

## 3. Results

### 3.1. Process of Selecting the Markers Evaluated in the EROS Study

**Figure 2:**
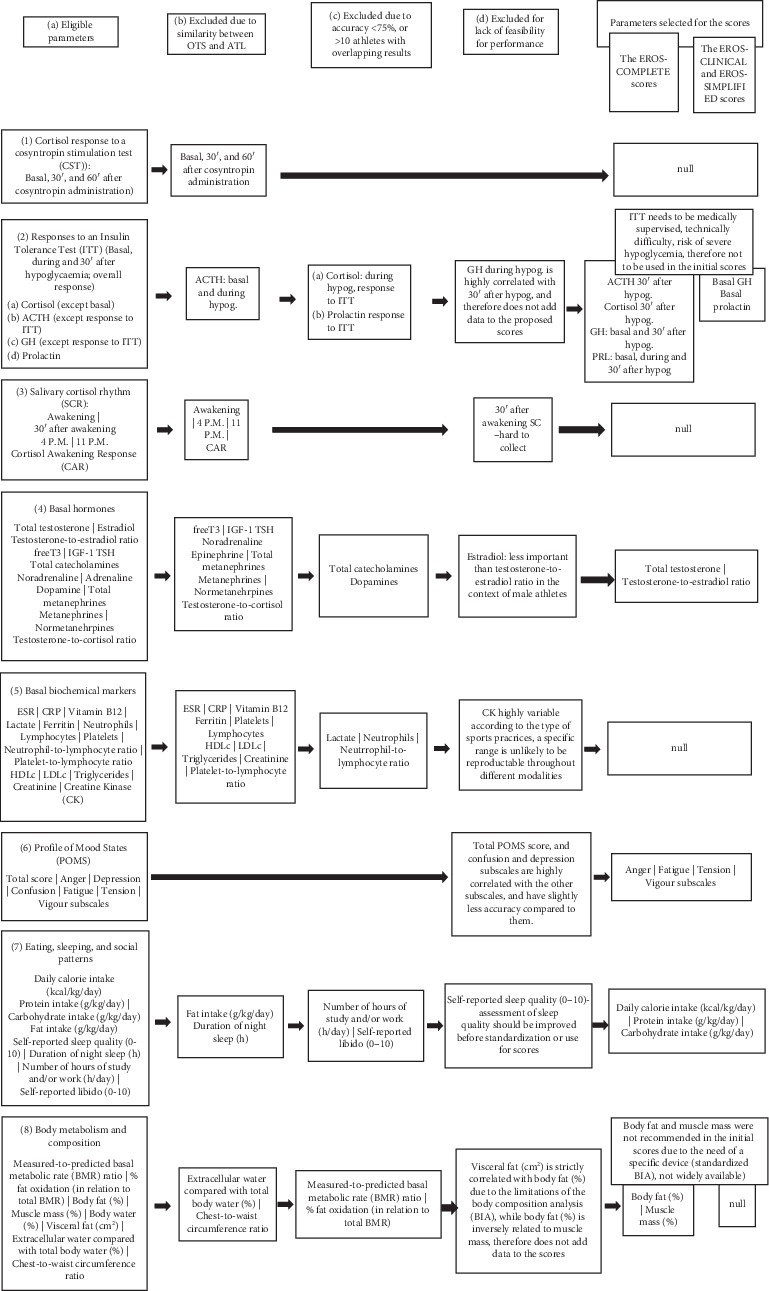
Selection process for choosing parameters for the proposed tools.

The analyses of statistical significance, accuracy, and number of overlapping results between the OTS and ATL groups for clinical and biochemical parameters are presented in Tables 3 and 4, respectively. The selection process for choosing the parameters in the present analysis is shown in Figure 2.

A total of 23 clinical and 50 biochemical parameters evaluated in the EROS study were examined for the development of the scores. Four clinical and 29 biochemical markers were excluded because they revealed similar results between the OTS and ATL groups. In addition, four clinical and eight biochemical parameters were excluded because of low accuracy or an excessive number of overlapping results between the OTS and ATL groups. Among the 15 clinical and 13 biochemical parameters that fulfilled the selection criteria, six clinical and four biochemical parameters were eliminated because their reproducibility was difficult, or they were inherently correlated with another selected parameter. We eventually selected nine biochemical and nine clinical parameters for the proposed scores (comprising 18 parameters). The selected parameters included four subscales (anger, fatigue, tension, and vigour) of the Profile of Mood States (POMS) questionnaire, three eating patterns (daily calorie intake [kcal/kg/day], protein intake [g/kg/day], and carbohydrate intake [kcal/kg/day]), and two characteristics of body composition (body fat [%] and muscle mass [%]), comprising nine clinical parameters. Also included were four basal hormones (GH, prolactin, total testosterone, and the testosterone-to-estradiol ratio) and five hormonal responses to an insulin tolerance test (ITT) (cortisol, ACTH, GH 30 minutes after hypoglycemia, and prolactin during and 30 minutes after hypoglycemia), comprising nine biochemical parameters. The ITT is the gold standard for evaluating the responsiveness of three hypothalamic–pituitary axes (somatotropic, corticotropic, and lactotropic axes) and is independent of physical performance, which eliminates underperformance bias found in OTS-affected athletes when compared to healthy athletes. For the EROS-RISK score, we also included self-reports of sleep quality (on a scale from 0 to 10, without the need for resource-intensive tests), the total POMS score, and the POMS depression and confusion subscales.

### 3.2. Combinations That Yielded 100% Accuracy in Distinguishing OTS from Healthy States

**Figure 3:**
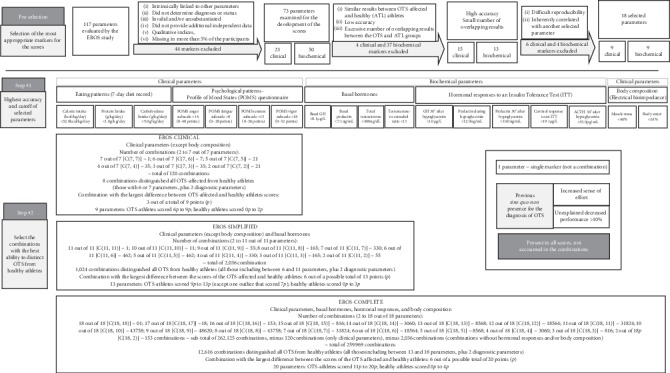
Flowchart for choosing the most suitable tool for use in clinical practice.

Using the 18 selected parameters, we tested 262,125 combinations to diagnose OTS, including 120 combinations with only clinical parameters (expect body composition), 2,036 with clinical and basal biochemical parameters (without stimulated hormones and body composition), and 259,969 with clinical, biochemical, and at least one stimulated hormone and/or body composition parameter. Of the 262,125 combinations, 13,648 distinguished all OTS-affected athletes from healthy athletes, including eight clinical-only parameters, 1,024 combined clinical and basal biochemical parameters, and 12,616 combined clinical, basal biochemical, and stimulated hormone parameters, which showed that OTS can be diagnosed using different combinations of biomarkers of OTS. The number of attempted combinations of parameters and the selection process for each score are detailed in Figure 3.

We proposed three different diagnostic tools, one from each cluster of combinations. Drawing from combinations with only clinical parameters (the EROS-CLINICAL combinations), we selected the combination with all nine clinical parameters for the present study because it detected the largest difference (3 points out of a possible total of 9 points) between the scores of the OTS-affected athletes and healthy athletes. Furthermore, these markers were simple to test and did not require the use of any device or technology. The proposed diagnostic tool is presented in Table 5.

Among the combinations that included the clinical and basal biochemical parameters, the one we selected as the EROS-SIMPLIFIED diagnostic tool included all nine clinical parameters and four basal biochemical parameters, for a total of 13 parameters, which yielded a 6-point (out of 13 points) difference between the scores of the OTS-affected and healthy athletes (with one outlier). The parameters included in this combination were also simple to test, as no bioelectrical impedance device or any other device was needed. The set of parameters for the EROS-SIMPLIFIED, a diagnostic tool, are shown in Table 6.

The EROS-COMPLETE set of parameters that combined clinical, basal, and stimulated hormones included 11 clinical parameters, of which nine were clinical parameters of the EROS-CLINICAL tool, plus two body-composition parameters and nine biochemical parameters, including four basal and five hormonal responses to an ITT, for a total of 20 parameters, as presented in Table 7. The selected combination yielded a difference of 6 points (out of a total of 20 points), between the scores of the OTS-affected and unaffected athletes.

The EROS-RISK, a diagnostic instrument, which is intended to address the prevention of OTS in high-risk athletes, included four of the five major risk factors of OTS, which were identified in the EROS study: carbohydrate, protein, and caloric intake—each one independent of the other, and inadequate sleep quality, and total and subscales of the POMS questionnaire, since the active self-perception of feelings and fatigue are underestimated by many athletes, as they tend to avoid perceptions of potential barriers to their trainings [12–14]. Although excessive concurrent working, studying, or any cognitive effort has also been identified as a risk factor for OTS, it failed to help identify those at risk for OTS [12–14]. The EROS-RISK is presented in Table 8.

## 4. Discussion

### 4.1. Diagnostic Tools for OTS

In contrast to the current shortage of diagnostic tools for OTS, different diagnostic tools were proposed in the present study, using combinations of highly accurate clinical and/or biochemical parameters. Some of the selected combinations of biomarkers identified for OTS and all three proposed diagnostic tools were found to be effective for diagnosing OTS with 100% accuracy in this sample of athletes. The diagnosis of OTS using these tools did not require the exclusion of confounding conditions [1], were easy to use, and did not require highly specialized sports personnel, indicating the fact that the proposed tools for diagnosing OTS were more user-friendly than the diagnostic flowchart recommended in the latest guidelines [1].

Although single markers were unable to distinguish OTS-affected athletes from healthy athletes, because of the high heterogeneity of OTS among the individual athletes, the relatively large number of true and naturally occurring OTS-affected athletes (the largest number among the studies on OTS [1–4]) and the large number of parameters evaluated in the EROS study facilitated the identification of more than 45 novel OTS biomarkers in that study. This enabled the evaluation of a possible diagnosis of OTS using combinations of parameters, among which more than 240,000 were tested. Thus, OTS was precisely identified in all affected athletes, while excluding OTS in healthy athletes, without the need to exclude confounding disorders in more than 4,000 different combinations, as many of the identified markers yielded few overlapping results between the OTS and ATL groups.

With respect to the two additional criteria included in all the proposed tools, although decreased performance and sense of increased effort to achieve the same level of training were the initial hallmarks and *sine-quo-non* criteria of OTS, they might appear only in the more severe stages of OTS, and progress unnoticed without proactive surveillance of athletes. At this stage, recovery is challenging and not always complete. Therefore, the identification of OTS at earlier stages is highly desirable, as recovery should be easier, faster, and less likely to compromise a training program. At this point, the presentation of OTS may be considered “unusual” because of the absence of a prolonged decline in performance, which is more likely to be underreported at this stage.

The diagnostic tools proposed in the present study made the possibility of diagnosing OTS feasible, as the presence of an unexplained decline in performance and increased sense of effort were optional and would strengthen the diagnosis of OTS but were not required. However, some authors would consider the existence of OTS impossible without a decline in performance because fatigue and underperformance would be likely to appear in milder forms of OTS, including functional and nonfunctional overreaching, while a complete recovery would avoid the development of OTS. However, the false perception of full recovery from these earlier stages, which is common because of the high motivation to return to the training sessions, could hide an underlying process that could ultimately lead to OTS. In addition, the belief that overreaching happens in the very early stage of OTS might not be true, as minor symptoms in the early stage are usually ignored by athletes. Finally, although many authors consider overreaching and overtraining as different stages of the same process, others claim that the underlying mechanisms between these states differ, as OTS tends to result from a more chronic, deeper, and central process, while overreaching tends to be characterized as an acute physical process. Therefore, our proposed diagnostic tools could be helpful to identify athletes prior to clinical signs of OTS with true underperformance, regardless of previous episodes of overreaching.

The EROS-CLINICAL is a diagnostic tool based on a combination of nine clinical parameters to yield a clinical score for athletes suspected of OTS, without the need to conduct biochemical tests. Our choice to exclude body fat and muscle mass as clinical parameters did not lead to any loss of information or reduction in the quality of the diagnostic assessment because not all facilities have available devices to analyze body composition, thereby precluding its clinical application, which is contrary to the main purpose of this diagnostic tool.

When the EROS-CLINICAL was inconclusive, the EROS-SIMPLIFIED with its combination of four additional basal hormones was successful in identifying most of the affected athletes not identified by the EROS-CLINICAL. Moreover, although the EROS-SIMPLIFIED tool includes biochemical parameters, it is simple to use, as functional tests are unnecessary.

The EROS-COMPLETE is a more comprehensive diagnostic tool encompassing the combination of all 18 selected parameters plus two inclusion criteria for OTS. It is valuable for (1) population-based screenings, irrespective of the presence of risks or suspected features of OTS, as OTS presentations are highly diverse; (2) research purposes, as it includes current biomarkers of OTS and can be used for comparisons with potential markers or assessment methods to identify new tools or improve our understanding of OTS; (3) unusual presentations of OTS that might remain undiagnosed if the guidelines with the diagnostic flowchart are used, as it provides a broad variety of markers, covering the full spectrum of OTS presentations; (4) exceptions, for an individual-based diagnostic approach, when the EROS-CLINICAL and EROS-SIMPLIFIED fail to diagnose OTS; and (5) understanding the unique underlying pathophysiology of each OTS-affected athlete.

In the EROS study, triggers of OTS other than excessive training were identified [5–8]. Therefore, athletes at high risk for OTS are not only those who undergo excessive training without adequate rest or athletes who show rapid progression in training volume or intensity. They also include athletes who (1) undergo long-term extreme diets, such as low carbohydrate, very low caloric intake, or a combination of intermittent fasting and calorie-restricted diets, without a compensatory reduction of training load, which happens to be an insufficient (but not hypocaloric) caloric, protein, and carbohydrate intake; (2) work or study excessively in an unceasing stressful environment, leading to concurrent cognitive and physical efforts that are uncompensated; or (3) have poor sleep hygiene and quality, usually related to the inability to disconnect from social media or TV.

Unfortunately, no single risk factor was able to identify all athletes at high risk. However, a combination of some modifiable risk factors invariably led to OTS when analyzed collectively. Given the lack of other tools to detect athletes at high risk for OTS, the EROS-RISK can be a helpful tool to identify these athletes and prevent the occurrence of OTS. At the prevention level, OTS can be addressed more effectively than it can at the treatment level. The POMS subscales may predict future occurrences of OTS, and for this reason, all of the POMS subscales are present in the EROS-RISK tool.

### 4.2. Limitations

Despite their 100% accuracy for the diagnosis of OTS, further tests of the proposed tools with larger populations are needed. The results of other studies may be inconclusive, as clinical presentations of OTS vary widely and the markers identified in our sample of athletes might not always be present. However, when the diagnosis of OTS is inconclusive using the EROS-CLINICAL, two additional diagnostic tools (the EROS-SIMPLIFIED and EROS-COMPLETE) can offer answers.

Despite the potential use of the diagnostic assessments recommended in the present study, they should be reproduced by further studies to strengthen and amend them as needed. They should not be considered as the gold standard of diagnostic tools for OTS at present, but rather, as an additional instrument.

Only male athletes that practiced both endurance and strength sports were evaluated. Therefore, whether these markers are present in other populations is unknown and should be addressed in future studies.

### 4.3. Final Discussion

**Figure 4:**
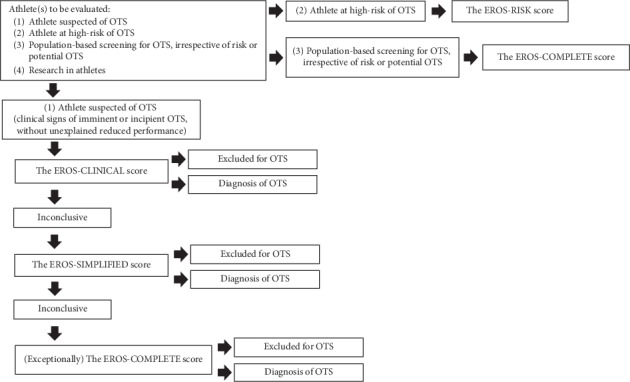
Flowchart for the choice of the most appropriate score to be used in each athlete.

To the best of our knowledge, these are the first diagnostic tools for OTS to achieve 100% accuracy in distinguishing OTS-affected athletes from healthy athletes without the need to exclude confounding disorders, or the need to include the presence of decreased performance. Furthermore, the tools are easy to use and are not time-consuming. The diagnostic tools proposed in the present study were made possible through the selection of parameters after more than 260,000 combination attempts. This is also the first study to suggest a preventive approach to OTS through an active search of forthcoming OTS in athletes at high risk, which has not been addressed by the current guidelines for OTS [1]. For clinical practice, Figure 4 displays a flowchart for the choice of the most appropriate score to be used in each athlete.

Although the proposed tools should not be the initial assessment for larger populations of athletes suspected of having OTS until they are reproduced and validated by further studies, they can be used in exceptional situations when the guidelines' flowchart is inconclusive or misleading.

The most important message of the present study is that while single markers did not diagnose OTS, combinations of OTS markers were found to be a feasible way to diagnose OTS accurately, in contrast to previous diagnostic tools. Further studies are needed to validate our findings and improve the tools' scoring systems by testing combinations of markers. This diagnostic approach corresponds to the major feature of OTS: its unique combination of alterations in each affected athlete.

## 5. Conclusion

In the present study, innovative tools were proposed for the diagnosis and prevention of OTS that yielded 100% accuracy in distinguishing overtraining syndrome from healthy states. This was done without the need to include the presence of decreased performance or exclude confounding disorders in this sample of athletes. These diagnostic approaches should be reproduced and validated as optional assessment tools for the diagnosis of OTS. Although OTS is highly heterogeneous, a combination of markers rather than a single marker appears to be more appropriate for the diagnosis of OTS, regardless of the proposed method.

## 6. Geological Information

All the research, including the participants enrolled and collection of the data, was conducted in Brasilia, Brazil, while the analysis of the results and development of the present manuscript were performed in São Paulo, Brazil.

## Figures and Tables

**Table 1:** Markers evaluated by the EROS study and included for the selection of the present analysis (aside from inclusion criteria for all groups: body mass index, age, and sex).

Study/tests	Markers
*EROS-HPA axis*	*Total number of markers: 73 (total number of evaluated markers: 14)*
Basal ACTH and cortisol and their response to an insulin tolerance test (ITT)	(1) Basal ACTH (pg/mL) and (2) cortisol (*µ*g/dL)
	(3) ACTH and (4) cortisol during hypoglycemia
	(5) ACTH and (6) cortisol 30 min after hypoglycemia
	(7) Cortisol increase during ITT
Cortisol response to a cosyntropin stimulation test (CST)	(8) Cortisol at 30 min and (9) at 60 min after injection
Salivary cortisol rhythm (SCR)	(10) Salivary cortisol (ng/dL) at awakening and (11) 30 min later
	(12) At 4 PM and (13) at 11 PM
	(14) Cortisol awakening response (CAR)

*EROS-Stress*	*(Total number of evaluated markers: 7)*
GH and prolactin response to an ITT	(1) Basal (GH) (*µ*g/L) and (2) prolactin (ng/mL)
	(3) GH and (4) prolactin during hypoglycemia
	(5) GH and (6) prolactin 30 min after hypoglycemia
	(7) Prolactin increase during ITT
Glucose behavior and related symptoms during an ITT (not included for the analysis)	Basal fasting glucose (mg/dL), glucose during hypoglycemia (mg/dL), time to hypoglycemia (min), adrenergic symptoms (0–10), and neuroglycopenic symptoms (0–10)

*EROS-Basal*	*(Total number of evaluated markers: 30)*
Hormonal markers	(1) Total testosterone (ng/dL) and (2) estradiol (pg/mL)
	(3) IGF-1 (pg/mL), (4) TSH (*µ*UI/mL), and (5) free T3 (pg/mL)
	(6) Total catecholamines and (7) metanephrines (both *µ*g/12 h)
	(8) Noradrenaline, (9) epinephrine, and (10) dopamine (all *µ*g/12 h)
	(11) Metanephrines and (12) normetanephrines (both *µ*g/12 h)
Biochemical markers	(13) Erythrocyte sedimentation rate (ESR, mm/h) and (14) creatine (mg/dL)
	(15) C-reactive protein (CRP, mg/dL) and (16) lactate (nMol/L)
	(17) Vitamin B12 (pg/mL) and (18) ferritin (ng/mL)
	(19) Neutrophils, (20) lymphocyte, and (21) eosinophils (all/mm^3^)
	(22) Creatine kinase (CK, U/L), (23) LDLc (mg/dL), (24) HDLc (mg/dL), (25) triglycerides (mg/dL), and (26) platelets (^*∗*^1000/mm^3^)
Ratios	(27) Testosterone-to-estradiol and (28) testosterone-to-cortisol ratios
	(29) Neutrophil-to-lymphocyte and (30) platelet-to-lymphocyte ratios

*EROS-PROFILE*	*(Total number of evaluated markers: 22)*
General patterns	(1) Duration of night sleep (h) and (2) self-reported sleep quality (0–10)
	(3) Self-reported libido (0–10) and (4) number of hours of activities (h/day)
Eating patterns	(5) Caloric intake (kcal/kg/day), (6) carbohydrate intake (g/kg/day), (7) protein intake (g/kg/day), and (8) fat intake (g/kg/day)
Psychological patterns	(9) Profile of Mood State (POMS) questionnaire (total score: −32 to +120)
	(10) Anger (0 to 48) and (6) confusion subscales (0 to 28)
	(11) Depression (0 to 60) and (12) vigour subscales (0 to 32)
	(13) Fatigue (0 to 28) and (14) tension subscales (0 to 36)
Body metabolism analysis	(15) Measured-to-predicted basal metabolic rate (BMR, %)
	(16) Percentage of fat burning compared to total BMR (%)
Body composition	(17) Body fat percentage (%) and (18) muscle mass weight (kg)
	(19) Body water percentage (BW, %) and (20) extracellular water compared to total BW (%)
	(21) Visceral fat (cm^2^)
	(22) Chest-to-waist circumference

**Table 2:** Clinical parameters evaluated for the development of the Endocrine and Metabolic Responses on Overtraining Syndrome diagnostic tools.

Study/tests	Markers	*p*-score	Highest NPV or PPV (%) and accuracy (%)	Ranges of the maximum accuracy and maximum true positive and true negative values	Useful for any of the questionnaires (no/potentially/yes)
EROS-PROFILE					

Eating patterns	Calorie intake (kcal/kg/day)	<0.001	Cutoff: 32–40 kcal/kg/day; PPV: 100%; NPV: 96.1%; accuracy: 97.4%	OTS: 13/14 if <32 kcal/kg/day; ATL: 25/25 if >40 kcal/kg/day or 22/25 if >47 kcal/kg/day; accuracy: 38/39 if 32–40 kcal/kg/day	Yes
	Protein intake (g/kg/day)	<0.001	Cutoff: 1.7 g/kg/day; PPV: 92.9%; NPV: 85.7%; accuracy: 84.6%	OTS: 14/14 if <2.5 g/kg/day, 13/14 if <2.2 g/kg/day or 10/14 if <1.6 g/kg/day; ATL: 24/25 if >1.6 g/kg/day or 19/25 if >2.5 g/kg/day; accuracy: 33/39 if 1.7 g/kg/day	Yes
	Carbohydrate intake (g/kg/day)	0.003	Cutoff: 5–5.4 g/kcal/day; PPV: 70%; NPV: 100%; accuracy: 84.6%	OTS: 14/14 if <5 g/kg/day or 10/14 if <3.2 g/kg/day; ATL: 23/25 if <3 g/kg/day or 18/25 if >6.5 g/kg/day; accuracy: 33/39 if 5–5.4 g/kg/day	Yes
	Fat intake (g/kg/day)	n/s	n/a	n/a	No

Social patterns	Self-reported sleep quality (0–10)	0.004	Cutoff: ≤5; PPV: 85.7%; NPV: 70.4%; accuracy: 82.1%	OTS: 12/14 if >8; ATL: 18/25 if <7; accuracy: 32/39 if ≤5	Potentially
	Duration of night sleep (h)	n/s	n/a	n/a	No
	Number of hours of activities (h/day)	<0.001	Cutoff: ≥9 h; PPV: 83.3%; NPV: 72.7%; accuracy: 74.4%	OTS: 10/14 if >7 h/day or 14/14 if >5 h/day; ATL: 25/25 if ≤10 h/day or 24/25 if ≤8 h/day; accuracy: 29/39 if ≥9 h/day	Potentially
	Self-reported libido (0–10)	0.024	Cutoff: ≤5; PPV: 66.7%; NPV: 73.3%; accuracy: 71.8%	OTS: 9/14 if <7 or 11/14 if <8; ATL: 22/25 if >5 or 20/25 if >6; accuracy: 28/39 if ≤5	Potentially

Psychological patterns	Total POMS questionnaire score (−32 to 120)	<0.001	Cutoff: 24–30; PPV: 100%; NPV: 89.3%; accuracy: 92.3%	OTS: 14/14 if >6, 13/14 if >18, or 11/14 if >24–30; ATL: 25/25 if <24 or 20/25 if <2; accuracy: 36/39 if 24–30	Yes
	POMS anger subscale (0–48)	0.003	Cutoff: ≥11; PPV: 81.8%; NPV: 82.2%; accuracy: 82.1%	OTS: 13/14 if >19 or 9/14 if >11; ATL: 23/25 if <11 or 19/25 if <8; accuracy: 32/39 if ≥11	Yes
	POMS confusion subscale (0–28)	0.001	Cutoff: ≥6; PPV: 85.7%; NPV: 75%; accuracy: 76.9%	OTS: 10/14 if >4 or 6/14 if >6; ATL: 24/25 if <6; accuracy: 30/39 if >6	Yes
	POMS depression subscale (0–60)	0.008	Cutoff: ≥9; PPV: 85.7%; NPV: 73.3%; accuracy: 76.9%	OTS: 6/14 if >9 or 8/14 if >6; ATL: 22/25 if <9 or 21/25 if <6; accuracy: 30/39 if <9	Yes
	POMS fatigue subscale (0–28)	<0.001	Cutoff: 5–7; PPV: 100%; NPV: 100%; accuracy: 100%	OTS: 14/14 if >8 or 12/14 if >13; ATL: 25/25 if <5; accuracy: 39/39 if 5–8	Yes
	POMS tension subscale (0–36)	<0.001	Cutoff: ≥13; PPV: 84.6%; NPV: 88.5%; accuracy: 87.2%	OTS: 14/14 if >5 or 11/14 if >13; ATL: 23/25 if <13; accuracy: 34/39 if 12 or 13	Yes
	POMS vigour subscale (0–32)	<0.001	Cutoff: ≤18; PPV: 100%; NPV: 96.2%; accuracy: 97.4%	OTS: 13/14 if <18; ATL: 25/25 if >18, 25/25 if >20, or 23/25 if >23; accuracy: 38/39 if >18–20	Yes

Body metabolism	Measured:predicted BMR (%)	0.013	Cutoff: <102%; PPV: 62.5%; NPV: 82.6%; accuracy: 74.4%	OTS: 13/14 if <108% or 10/14 if <102%; ATL: 19/25 if >102%; accuracy: 29/39 if 102%	Potentially
	Percentage of fat burned compared with total BMR (%)	<0.001	Cutoff: <38%; PPV: 75%; NPV: 74.2%; accuracy: 74.4%; good for PPV and NPV; not good for accuracy	OTS: 14/14 if <58% or 6/14 if <40% and <30%; ATL: 23/25 if >20% or >38%; accuracy: 29/39 if <30–38%	Potentially

Body composition	Body fat (%)	<0.001	Cutoff: >17%; PPV: 80%; NPV: 70.6%; accuracy: 76.9%	OTS: 14/14 if >10%, 7/14 if >15% or 4/14 if >17%; ATL: 24/25 if <17% or 22/25 if <15%; accuracy: 30/39 if >17%	Yes
	Muscle mass (%)	0.008	Cutoff: <47%; PPV: 100%; NPV: 75.8%; accuracy: 79.5%	OTS: 12/14 if <50% or 6/14 if <47%; ATL: 25/25 if >47%; accuracy: 31/39 if 47%	Yes
	Body water (%)	<0.001	Cutoff: <60%; PPV: 100%; NPV: 75.8%; accuracy: 79.5%	OTS: 14/14 if <65%, 13/14 if <63.5% or 6/14 if <60%; ATL: 25/25 if >60%; accuracy: 31/39 if 60%	Yes
	Extracellular water (%)	n/s	n/a	n/a	No
	Visceral fat (cm^2^)	0.01	Cutoff: >68 cm^2^: PPV: 87.5%; NPV: 77.4%; accuracy: 79.5%	OTS: 13/14 if >38 cm^2^ or 7/14 if >68 cm^2^; ATL: 24/25 if <68 cm^2^, 22/25 if <56 cm^2^, or 14/25 if <38 cm^2^	Potentially
	Chest-to-waist circumference ratio	n/s	n/a	n/a	No

ATL = healthy athletes; BMR = basal metabolic rate; EROS = Endocrine and Metabolic Responses on Overtraining Syndrome; n/a = nonapplicable; NPV = negative predictive value; n/s = nonsignificant; OTS = overtraining syndrome-affected athletes; POMS = Profile of Mood States; PPV = positive predictive value. ^*∗*^Not included for the analysis.

**Table 3:** Biochemical parameters evaluated for the development of the Endocrine and Metabolic Responses on Overtraining Syndrome diagnostic tools.

Study/tests	Markers	*p*-score	Highest NPV or PPV (%) and accuracy (%)	Ranges of the maximum accuracy and maximum true positive and true negative values	Useful for any of the questionnaires (no/potentially/yes)
*EROS-HPA axis*					
Response to an ITT	Basal ACTH levels (pg/mL)	n/s	n/a	n/a	No
	ACTH levels during hypoglycemia (pg/mL)	n/s	n/a	n/a	No
	ACTH levels 30 min after hypoglycemia (pg/mL)	<0.001	Cutoff: <35 pg/mL; PPV: 75%; NPV: 81.5%; accuracy: 79.5%	OTS: 13/14 if <106 pg/mL or 9/14 if <35 pg/mL; ATL: 22/25 if >35 pg/mL; accuracy: 31/39 if >35 pg/mL	Yes
	ACTH increase during an ITT (pg/mL)^*∗*^	<0.001	Cutoff: <20 pg/mL; PPV: 66.7%; NPV: 83.3%; accuracy: 76.9%	OTS: 14/14 if <75 pg/mL, 12/14 if <35 pg/mL, or 10/14 if <20 pg/mL; ATL: 25/25 if >3 pg/mL or 20/25 if >20 pg/mL; accuracy: 30/39 if 1–3 pg/mL or 20 pg/mL	Potentially
	Basal serum cortisol levels (*µ*g/dL)	n/s	n/a	n/a	n/a
	Cortisol levels during hypoglycemia (*µ*g/dL)	0.015	Cutoff: <13.5 μg/dL; PPV: 62.5%; NPV: 82.6%; accuracy: 74.4%	OTS: 12/14 if <16.9 *µ*g/dL, 11/14 if <13.7 *µ*g/dL, or 10/14 if <13.2 *µ*g/dL; ATL: 17/25 if >13.7 *µ*g/dL or 19/25 if >13.5 *µ*g/dL; accuracy: 29/39 if 13.2–13.5 *µ*g/dL	No (10/39 overlapping results)
	Cortisol levels 30 min after hypoglycemia (*µ*g/dL)	0.002	Cutoff: <19.1 *µ*g/dL; PPV: 72.2%; NPV: 95.2%; accuracy: 84.6%	OTS: 13/14 if <19.1 *µ*g/dL; ATL: 25/25 if <17 *µ*g/dL or 20/25 if <19.1 *µ*g/dL; accuracy: 33/39 if <19.1 *µ*g/dL	Yes
	Cortisol increase during an ITT (*µ*g/dL)	0.008	Cutoff: <9 *µ*g/dL; PPV: 57.1%; NPV: 89.9%; accuracy: 71.8%	OTS: 14/14 if <10 *µ*g/dL, 12/14 if <9 *µ*g/dL, or 11/14 if <8 *µ*g/dL; ATL: 16/25 if >9 *µ*g/dL, 13/25 if >10 *µ*g/dL, or 10/25 if >11 *µ*g/dL; accuracy: 28/39 if <9 *µ*g/dL	No (11/39 overlapping results)

Response to a CST	Cortisol levels 30 min after cosyntropin (*µ*g/dL)	n/s	n/a	n/a	No
	Cortisol levels 60 min after cosyntropin (*µ*g/dL)	n/s	n/a	n/a	No

SCR	Waking salivary cortisol (ng/dL)	n/s	n/a	n/a	No
	Salivary cortisol 30 min after waking (ng/dL)	0.002	Cutoff: >390 ng/dL; PPV: 68.7%; NPV: 87%; accuracy: 79.5%	OTS: 14/14 if <520 ng/dL or 11/14 if <390 ng/dL; ATL: 20/25 if >390–400 ng/dL; Accuracy: 31/39 if <390–400 ng/dL	Yes
	4 PM salivary cortisol (ng/dL)	n/s	n/a	n/a	No
	11 PM salivary cortisol (ng/dL)	n/s	n/a	n/a	No
	CAR (%)	n/s	n/a	n/a	No
*EROS-STRESS*					
Response to an ITT	Basal GH levels (*µ*g/L)	0.009	Cutoff: <0.1 *µ*g/L; PPV: 87.5%; NPV: 77.4%; accuracy: 79.5%	OTS: 11/14 if <0.2 *µ*g/L or 7/14 <0.07 *µ*g/L; ATL: 24/25 if >0.1 *µ*g/L or 18/25 if >0.2 *µ*g/L; Accuracy: 31/39 if <0.1 *µ*g/L	Yes
	GH levels during hypoglycemia (*µ*g/L)	0.018	Cutoff: <0.1 *µ*g/L; PPV: 100%; NPV: 73.5%; accuracy: 76.9%	OTS: 12/14 if <1.8 *µ*g/L, 8/14 if <0.7 *µ*g/L, 6/14 if <0.4 *µ*g/L, or 5/14 if <0.25 *µ*g/L; ATL: 25/25 if >0.1 *µ*g/L, 24/25 if >0.17 *µ*g/L, 22/25 if >0.3 *µ*g/L, 18/25 if >0.4 *µ*g/L, or 16/25 if >0.5 *µ*g/L; accuracy: 30/39 if <0.1 *µ*g/L	Yes
	GH levels 30 min after hypoglycemia (*µ*g/L)	0.001	Cutoff: <5–6 *µ*g/L; PPV: 81.8%; NPV: 82.1%; accuracy: 82%	OTS: 14/14 if <14.4 *µ*g/L, 12/14 if <10.7 *µ*g/L, 9/14 if <6 *µ*g/L, or 7/14 if <1.5 *µ*g/L; ATL: 23/25 if >6 *µ*g/L; accuracy: 32/39 if <1.5–560 *µ*g/L	Potentially
	Basal prolactin levels (ng/mL)	0.014	Cutoff: <7.1 ng/mL; PPV: 85.7%; NPV: 75%; accuracy: 76.9%	OTS: 13/14 <14.5 ng/mL or 6/14 if <7.1 ng/mL; ATL: 25/25 if >6.6 ng/mL, 24/25 if >7.1 ng/mL, or 23/25 if >7.7 ng/mL; accuracy: 30/39 if <6.6–7.7 ng/mL	Yes
	Prolactin levels during hypoglycemia (ng/mL)	0.001	Cutoff: <11.5–12 ng/mL; PPV: 83.3%; NPV: 85.2%; accuracy: 84.6%	OTS: 12/14 if <17.5 ng/mL or 10/14 if >11.5–12 ng/mL; ATL: 23/25 if <12 ng/mL; accuracy: 33/39 if <11.5–12 ng/mL	Yes
	Prolactin levels 30 min after hypoglycemia (ng/mL)	0.001	Cutoff: <10 ng/mL; PPV: 100%; NPV: 78.1%; accuracy: 82.1%	OTS: 7/14 if <14 ng/mL or <10 ng/mL; ATL: 25/25 if 10 ng/mL or 18/25 if >20 ng/mL; accuracy: 32/39 if <10 ng/mL	No (11/39 overlapping results)
	Prolactin increase during an ITT (ng/mL)	0.047	Cutoff: <0–2.5 ng/mL; PPV: 61.5%; NPV: 76.9%; accuracy: 71.8%	OTS: 14/14 if <14 ng/mL, 10/14 if <4 ng/mL, or 8/14 if <0 ng/mL; ATL: 20/25 if > 0–2.5 ng/mL; accuracy: 28/39 if <0–2.5 ng/mL	

Glucose behavior and related symptoms during an ITT	Basal serum glucose (mg/dL)	n/s	n/a	n/a	No
	Serum glucose during hypoglycemia (mg/dL)	n/s	n/a	n/a	No
	Time to hypoglycemia (min)	n/s	n/a	n/a	No
	Adrenergic symptoms (0–10)	0.034	Cutoff: ≤3: PPV: 56.2%; NPV: 78.3%; accuracy: 69.2%	OTS: 13/14 if ≤6 or 9/14 if ≤3; ATL: 20/25 if ≥3 or 18/25 if ≥4; accuracy: 27/39 if ≤3	No (12/39 overlapping results)
	Neuroglycopenic symptoms (0–10)	n/s	n/a	n/a	No
*EROS-BASAL*					
Hormonal markers	Total testosterone (ng/dL)	0.008	Cutoff: <400 pg/mL; PPV: 72.7%; NPV: 78.6%; accuracy: 76.9%	OTS: 10/14 if <440 pg/mL, 8/14 if <380 pg/mL; ATL: 24/25 if >350 ng/mL or 22/25 if >400 ng/mL; accuracy: 30/39 if <380–410 pg/mL	Potentially
	Estradiol (pg/mL)^*∗*^	0.007	Cutoff: >35 pg/mL; PPV: 66.7%; NPV: 75%; accuracy: 75.7%	OTS: 13/14 if >28 pg/mL or 10/14 if <35.5 pg/mL; ATL: 18/23 if <34 pg/mL; accuracy: 28/37 if >34–35.5 pg/mL^*∗*^	Potentially
	IGF-1 (pg/mL)	n/s	n/a	n/a	No
	TSH (*µ*UI/mL)	n/s	n/a	n/a	No
	free T3 (pg/mL)	n/s	n/a	n/a	No
	Total catecholamines (*µ*g/12 h)	0.032	Cutoff: >220 *µ*g/12 h; PPV: 58.3%; NPV: 74.1%; accuracy: 69.2%	OTS: 14/14 if >130 *µ*g/12 h, 11/14 if >155 *µ*g/12 h, 8/14 if >200 *µ*g/12 h, 7/14 if >220 *µ*g/12 h, or 5/14 if >270 *µ*g/12 h; ATL: 20/25 if <220 *µ*g/12 h, 15/25 if <200 *µ*g/12 h, 11/25 if <150 *µ*g/12 h, or 10/25 if <130 *µ*g/12 h; accuracy: 27/39 if >220 *µ*g/12 h	No (12/39 overlapping results)
	Noradrenaline (*µ*g/12 h)	n/s	n/a	n/a	No
	Dopamine (*µ*g/12 h)	0.042	Cutoff: >200 *µ*g/12 h; PPV: 58.3%; NPV: 74.1%; Accuracy: 69.2%	OTS: 12/14 if >120 *µ*g/12 h, 7/14 if >200 *µ*g/12 h, or 4/14 if >230 *µ*g/12 h; ATL: 20/25 if <200 *µ*g/12 h, 13/25 if <160 *µ*g/12 h, or 11/25 if <120 *µ*g/12 h; accuracy: 27/39 if >200 *µ*g/12 h	No (12/39 overlapping results)
	Epinephrine (*µ*g/12 h)	n/s	n/a	n/a	No
	Total metanephrines (*µ*g/12 h)	n/s	n/a	n/a	No
	Metanephrines (*µ*g/12 h)	n/s	n/a	n/a	No
	Normetanephrines (*µ*g/12 h)	n/s	n/a	n/a	No

Biochemical markers	ESR (mm/h)	n/s	n/a	n/a	No
	CRP (mg/dL)	n/s	n/a	n/a	No
	Vitamin B12 (pg/mL)	n/s	n/a	n/a	No
	Lactate (nMol/L)	0.007	Multiple cutoffs: >0.9, 0.92–0.93, 0.95–0.98, or 1.06–1.1 nMol/L; PVV: 77.8% if >1.08 nMol/L; NPV: 100% if <0.75 nMol/L; accuracy: 74.4%	OTS: 14/14 if >0.75 nMol/L; ATL: 21/25 if <1.05 nMol/L; accuracy: 29/39 if 0.9, 0.92–0.93, 0.95–0.98, or 1.06–1.1 nMol/L	No (10/39 overlapping results)
	Ferritin (ng/mL)	n/s	n/a	n/a	No
	Neutrophils (/mm^3^)^*∗∗*^	0.035	Cutoff: 2700/mm^3^; PPV: 61.5%; NPV: 76%; accuracy: 71.1%	OTS: 12/14 if <3500/mm^3^ or 8/14 if <2700/mm^3^; ATL: 19/24 if >2700/mm^3^ or 14/24 if >3500/mm^3^; accuracy: 27/38 if <2700/mm^3^, 26/38 if <2900/mm^3^, or 3500–3750/mm^3^	No (accuracy <75%)
	Lymphocytes (/mm^3^)	n/s	n/a	n/a	No
	Platelets (^*∗*^1000/mm^3^)	n/s	n/a	n/a	No
	Hematocrit (%)^*∗*^	n/s	n/a	n/a	No
	Eosinophils (/mm^3^)	n/s	n/a	n/a	No
	LDLc (mg/dL)	n/s	n/a	n/a	No
	HDLc (mg/dL)	n/s	n/a	n/a	No
	Triglycerides (mg/dL)	n/s	n/a	n/a	No
	CK (U/L)	0.043	Cutoff: 800–850 U/L; PPV: 85.7%; NPV: 75%; accuracy: 76.9%	OTS: 7/14 if >760 U/L or 6/14 if >880 U/L; ATL: 24/25 if <780–910 U/L or 22/25 if <580–730 U/L; accuracy: 30/39 if 730 U/L or between 800 and 850 U/L	Potentially
	Creatinine (mg/dL)	n/s	n/a	n/a	No

Ratios	Testosterone:estradiol ratio^*∗*^	<0.001	Cutoff: 13.3; PPV: 83.3%; NPV: 84%; accuracy: 83.8%	OTS: 14/14 if <17 or 10/14 if <13.3; ATL: 21/23 if >13 or 16/23 if >16; accuracy: 31/37 if 13.3	Yes
	Testosterone:cortisol ratio	n/s	n/a	n/a	No
	Neutrophil:lymphocyte ratio^*∗∗*^	0.017	Cutoff: <1.65; PPV: 54.2%; NPV: 92.3%; accuracy: 68.4%	OTS: 14/14 if <1.95 or 13/14 if <1.65; ATL: 13/24 if >1.65 or 11/24 if >1.95;	No (accuracy <75%)
	Platelet:lymphocyte ratio^*∗∗*^	n/s	n/a	accuracy: 26/38 if <1.65	No

^*∗*^Two missing data points. ^*∗∗*^One missing data point. ACTH = adrenocorticotropic hormone; ATL = healthy athletes; CAR = cortisol awakening response; CK = creatine kinase; CRP = C-reactive protein; CST = cosyntropin stimulation test; EROS = Endocrine and Metabolic Responses to Overtraining Syndrome; ESR = erythrocyte sedimentation rate; GH = growth hormone; HDLc = high-density lipoprotein-cholesterol; HPA = hypothalamic–pituitary–adrenal; IGF-1 = insulin-like growth factor 1; ITT = insulin tolerance test; LDLc = low-density lipoprotein-cholesterol; n/a = nonapplicable; NPV = negative predictive value; n/s = nonsignificant; OTS = overtraining syndrome-affected athletes; PPV = positive predictive value; SCR, salivary cortisol rhythm; T3L = free T3; and TSH = thyroid-stimulating hormone.

**Table 4:** Characteristics of the proposed diagnostic tools.

Tool	Target athletes	Aim	Number of parameters	Score (points) and criteria
EROS-CLINICAL	Suspected of OTS (possible signs of imminent or incipient OTS)	Diagnosis of OTS in suspected athletes, easy-to-perform and not time- or fund-consuming	9	0–2 = excluded for OTS 3–5 = inconclusive 6–9 = diagnosis of OTS

EROS-SIMPLIFIED	Suspected of OTS when the diagnosis was not confirmed using the EROS-CLINICAL criteria	Diagnosis of OTS in suspected athletes when the diagnosis was not confirmed using the EROS-CLINICAL criteria	13	0–3 = excluded for OTS 4–6 = inconclusive 7–8 = probable OTS 9–13 = diagnosis of OTS

EROS-COMPLETE	Population-based screenings; athletes participating in research Exception, suspected of OTS when the diagnosis was not confirmed using the EROS-SIMPLIFIED criteria	Diagnosis of OTS in large populations of athletes, irrespective of the risk or probability of OTS Identification of risk factors, biomarkers, and tools for the prevention and diagnosis of OTS Exception, individual diagnosis of OTS when the diagnosis was not confirmed using the EROS-SIMPLIFIED criteria	20	0–4 = excluded for OTS 5–10 = inconclusive 11–20 = diagnosis of OTS

EROS-RISK	At high risk for OTS (absence of clinical or biochemical signs)	Prevention of OTS in high-risk athletes	11	0–1 = low risk of OTS 2–4 = moderate risk of OTS 5–6 = high risk of OTS 7–11 = imminent risk of OTS

OTS = overtraining syndrome.

**Table 5:** The EROS-CLINICAL tool.

Risk factor	Range	Score (to be entered)^*∗*^
*(a) Scores with ranges*		
Calorie intake (kcal/kg/day)	<32.0	
Protein intake (g/kg/day)	<1.6	
Carbohydrate intake (g/kg/day)	<5.0	
POMS anger subscale (0–48 points)	>14	
POMS fatigue subscale (0–28 points)	>8	
POMS tension subscale (0–36 points)	>13	
POMS vigour subscale (0–32 points)	<18	
Unexplained decreased performance > 10%	Y/N	
Increased sense of effort	Y/N	
Total	0–9	

Score	Interpretation	
*(b) Interpretation of the results*		
0–2 points	Diagnosis excluded for overtraining syndrome	
3–5 points	Inconclusive (at intermediate-to-high risk for OTS, or an unusual presentation of OTS)	
6–9 points	Diagnosis of overtraining syndrome	

^*∗*^Each parameter within the range is assigned 1 point. POMS = Profile of Mood States; OTS = overtraining syndrome.

**Table 6:** The SIMPLIFIED-OTS diagnostic score.

RISK factor	Range	Points (to be entered)^*∗*^
*(a) Scores with ranges*		
Calorie intake (kcal/kg/day)	<32.0	
Protein intake (g/kg/day)	<1.6	
Carbohydrate intake (g/kg/day)	<5.0	
POMS anger subscale (0–48 points)	>14	
POMS fatigue subscale (0–28 points)	>8	
POMS tension subscale (0–36 points)	>13	
POMS vigour subscale (0–32 points)	<18	
Decreased performance > 10%	Yes	
Increased sense of effort	Yes	
Basal GH (*µ*g/L)	<0.1	
Basal prolactin (ng/mL)	<7.1	
Total testosterone (ng/dL)	<400	
Testosterone-to-estradiol ratio	<13	
Total (points)	0–13	

Score	Interpretation	
*(b) Interpretation of the results*		
0–3 points	Overtraining syndrome excluded	
4–6 points	Inconclusive (imminent, incipient, or unusual presentation of overtraining syndrome)	
7–13 points	Overtraining syndrome confirmed	

^*∗*^Each parameter within the range is assigned 1 point. POMS = Profile of Mood States; GH = growth hormone; and OTS = overtraining syndrome.

**Table 7:** The EROS-COMPLETE tool.

Risk factor	Range	Points (to be entered)^*∗*^
*(a) Scores with ranges.* ^*∗*^ *Each parameter within the range is assigned 1 point*		
Decreased performance >10%	Yes	
Increased sense of effort	Yes	
Calorie intake (kcal/kg/day)	<32.0	
Protein intake (g/kg/day)	<1.6	
Carbohydrate intake (g/kg/day)	<5.0	
POMS anger subscale (0–48)	>14	
POMS fatigue subscale (0–28)	>8	
POMS tension subscale (0–36)	>13	
POMS vigour subscale (0–32)	<18	
Muscle mass (%)	<46	
Body water (%)	<61	
ACTH 30 minutes after hypoglycemia (pg/mL)	<35	
Cortisol response to ITT (*µ*g/dL)	<19.1	
Basal GH (*µ*g/L)	<0.1	
GH 30 minutes after hypoglycemia (*µ*g/L)	<1.0	
Basal prolactin (ng/mL)	<7.1	
Prolactin during ITT (ng/mL)	<12	
Prolactin 30 minutes after hypoglycemia (ng/mL)	<10	
Total testosterone (ng/dL)	<400	
Testosterone to estradiol ratio	<13	
Total	0–20	

Score	Interpretation	
*(b) Interpretation of the results*		
0–4 points	Excluded diagnosis for overtraining syndrome	
5–10 points	Inconclusive (at intermediate-to-high risk for OTS, or an unusual presentation of OTS)	
11–20 points	Diagnosis of overtraining syndrome	

POMS = Profile of Mood States; ITT = insulin tolerance test; ACTH = Adrenocorticotropic hormone; GH = growth hormone; and OTS = overtraining syndrome.

**Table 8:** The OTS-RISK score for the assessment of risk level for the development of overtraining syndrome.

Risk factor	Range	Points (to be entered)^*∗∗*^
*(a) Score, with ranges and points*		
Calorie intake^*∗*^	<32 kcal/kg/day	
Protein intake^*∗*^	<1.6 g/kg/day	
Carbohydrate intake^*∗*^	<5 g/kg/day	
Total POMS score (−32 to 120)	>19	
POMS anger subscale (0–48)	>14	
POMS confusion subscale (0–28)	>6	
POMS depression subscale (0–60)	>7	
POMS fatigue subscale (0–28)	>8	
POMS tension subscale (0–36) >13	>13	
POMS vigour subscale (0–32) <18	<18	
Self-reported sleep quality (0–10)	<6 (≤5)	
Total	0–11	

Score (points)	Interpretation	
*(b) Interpretation of the results*		
0-1	Low risk of OTS	
2–4	Moderate risk of OTS	
5-6	High risk of OTS	
7–11	Imminent risk of OTS	

OTS = overtraining syndrome; POMS = Profile of Mood States. ^*∗*^Using a 7-day dietary record. ^*∗∗*^Each parameter within the range is assigned 1 point.

## Data Availability

The raw data of the present study are available at the repository https://osf.io/bhpq9/, and the supporting data are also available at the list of references [7–11].
